# Low body mass index demonstrates satisfactory specificity for diagnosing malnutrition and is associated with longer hospitalization in patients with gastrointestinal or head and neck cancer: a prospective cohort study

**DOI:** 10.3332/ecancer.2025.1846

**Published:** 2025-02-12

**Authors:** Camilla Horn Soares, Giovanna Potrick Stefani, Laura Machado Scott, Mariana Scortegagna Crestani, Thais Steemburgo

**Affiliations:** 1 Graduate Program in Food, Nutrition, and Health, Universidade Federal do Rio Grande do Sul, Porto Alegre 90010-150, Rio Grande do Sul, Brazil; 2 Hospital de Cl’nicas de Porto Alegre, Porto Alegre 90035-903, Rio Grande do Sul, Brazil; 3 Department of Nutrition, Universidade Federal do Rio Grande do Sul, Porto Alegre 90010-150, Rio Grande do Sul, Brazil; a https://orcid.org/0000-0002-5659-9660; b https://orcid.org/0000-0001-7469-0268; c https://orcid.org/0000-0002-7557-1786; d https://orcid.org/0000-0003-0526-7110; e https://orcid.org/0000-0003-3351-9901

**Keywords:** cancer, anthropometry, nutritional status, malnutrition, length of hospital stay

## Abstract

**Background:**

The main causes of malnutrition in patients with gastrointestinal and head and neck cancers include metabolic alterations determined by the tumour and its stage, as well as low food intake caused by the disease itself and the effects of antineoplastic treatment. In the hospital environment, nutritional markers, such as body mass index (BMI), handgrip strength (HGS) and calf circumference (CC), can be used to identify malnutrition early, ensuring individualized and specific nutritional intervention. However, few studies have evaluated the individual performance of nutritional indicators in diagnosing malnutrition in a cancer setting. We aimed to assess the ability of these nutritional indicators to accurately diagnose malnutrition and their association with length of hospital stay (LOS) in patients with cancer.

**Methods:**

This cohort study prospectively evaluated 171 patients with gastrointestinal or head and neck cancer. Nutritional status was assessed within 48 hours of hospital admission using BMI, CC and HGS as well as two reference standards: subjective global assessment (SGA) and patient-generated SGA (PG-SGA). The accuracy of each nutritional indicator was measured by the area under the receiver operating characteristic curve (AUC) compared with the reference standards. Multiple logistic regression analysis, adjusted for confounders, was used to determine whether malnutrition was associated with LOS.

**Results:**

Of 171 patients, 59.1% had low CC, 46.2% had low HGS and 13.5% had low BMI. The SGA and PG-SGA scores indicated malnutrition in 57.4% and 87.2% of patients, respectively. All nutritional indicators had poor accuracy in diagnosing malnutrition (AUC < 0.70). However, compared with SGA and PG-SGA, low BMI had satisfactory specificity (>80%) and was associated with 1.79 times higher odds of LOS ≥ 6 days. Malnutrition diagnosed by SGA and PG-SGA increased the odds of LOS ≥ 6 days by 3.60-fold and 2.78-fold, respectively.

**Conclusion:**

Low BMI showed adequate specificity for diagnosing malnutrition and was associated with longer LOS in patients with gastrointestinal or head and neck cancer. Further research is needed to explore how improved screening, interventions and nutritional support could reduce malnutrition rates in cancer patients.

## Introduction

In cancer settings, malnutrition is considered a risk factor for many complications such as increased hospital length of stay (LOS), hospital readmission, poor response to treatment and mortality [1–3]. Potential causes of malnutrition include cancer-related metabolic changes, genetic factors and environmental or care-related factors such as hospital nutrition [4, 5]. Patients with cancer often experience a change in metabolism due to the energy needs of growing tumours, which leads to muscle wasting, weight loss and inflammation [6]. Indeed, inflammatory cytokines (e.g., such as tumour necrosis factor-α and interleukin-6), are frequently elevated in cancer patients, contributing to anorexia and muscle wasting [7]. Genetic variations can also affect nutrient metabolism, potentially increasing the vulnerability of some cancer patients to malnutrition [8]. For instance, certain mutations may alter protein synthesis or energy metabolism in ways that worsen the body’s ability to maintain appropriate nutrition during cancer treatment [8]. In the hospital environment, the prevalence of malnutrition in individuals with cancer ranges from 35% to 71% depending on the diagnostic criteria used [9]. Therefore, hospital nutrition plays a significant role in the nutritional status of cancer patients [5]. However, maintaining adequate nutritional intake through a healthy, nutrient-rich diet remains a significant challenge for many patients undergoing cancer treatment [10]. In fact, a considerable number of patients with cancer who have experienced a notable decline in food intake frequently do not receive specific dietary counselling or any prescription of nutritional supplements to prevent or reduce weight loss during hospitalization [4, 5].

Involuntary weight loss can affect 50%–80% of patients with cancer and will vary in degree depending on the type, stage and location of the tumour [11]. In patients with gastrointestinal or head and neck cancer, both nutritional risk and malnutrition prevalence are increased due to nutritional deficits present in these types of cancer caused by the disease itself and the effects of antitumour treatment, which contribute to decreased nutrient intake [12–14]. In gastrointestinal tumours, mechanical obstruction can occur leading to nutrient malabsorption [15]. In head and neck tumours, the main symptoms are dysphagia, mucositis, difficulty chewing, odynophagia and decreased food intake [16].

Continuous nutrition monitoring is required in patients with cancer due to the close relationship between nutritional deficits and reduced response to antitumour therapy and decreased quality of life [17]. The validated tools for early detection of malnutrition in hospitalized patients with cancer include the subjective global assessment (SGA) and the patient-generated SGA (PG-SGA) [18]. SGA is the method of choice to assess nutritional status [19], where poor status is associated with increased LOS and mortality [20, 21]. PG-SGA is used in the cancer setting [22], and a PG-SGA diagnosis of malnutrition is also strongly associated with prolonged LOS [23, 24].

Nutritional indicators have been used as a complement to nutritional assessment in patients with cancer because of their relationship to malnutrition, including body mass index (BMI), calf circumference (CC) and handgrip strength (HGS) [25–29]. BMI most commonly categorizes patients into underweight, normal weight, overweight and obesity [30], where very low BMI (<18 kg/m^2^) has been associated with poor clinical outcomes, including an increased risk of death [31, 32]. CC measurement is strongly associated with skeletal muscle mass, serving as a useful predictor of hospital readmission and mortality in patients with cancer [27, 28, 33]. HGS has been used to assess muscle function and functional capacity [34, 35]. In patients with different types of cancer, those with low HGS were found to be three times less likely to be discharged from the hospital [23].

Because malnutrition is prevalent in patients with gastrointestinal or head and neck cancer, this study aimed to evaluate the ability of individual nutritional indicators (BMI, CC and HGS) to accurately diagnose malnutrition in these patients, using SGA and PG-SGA as the reference standard methods, and to examine potential associations of nutritional indicators and malnutrition with LOS as the outcome.

## Methods

### Study design and participants

**Figure 1.**
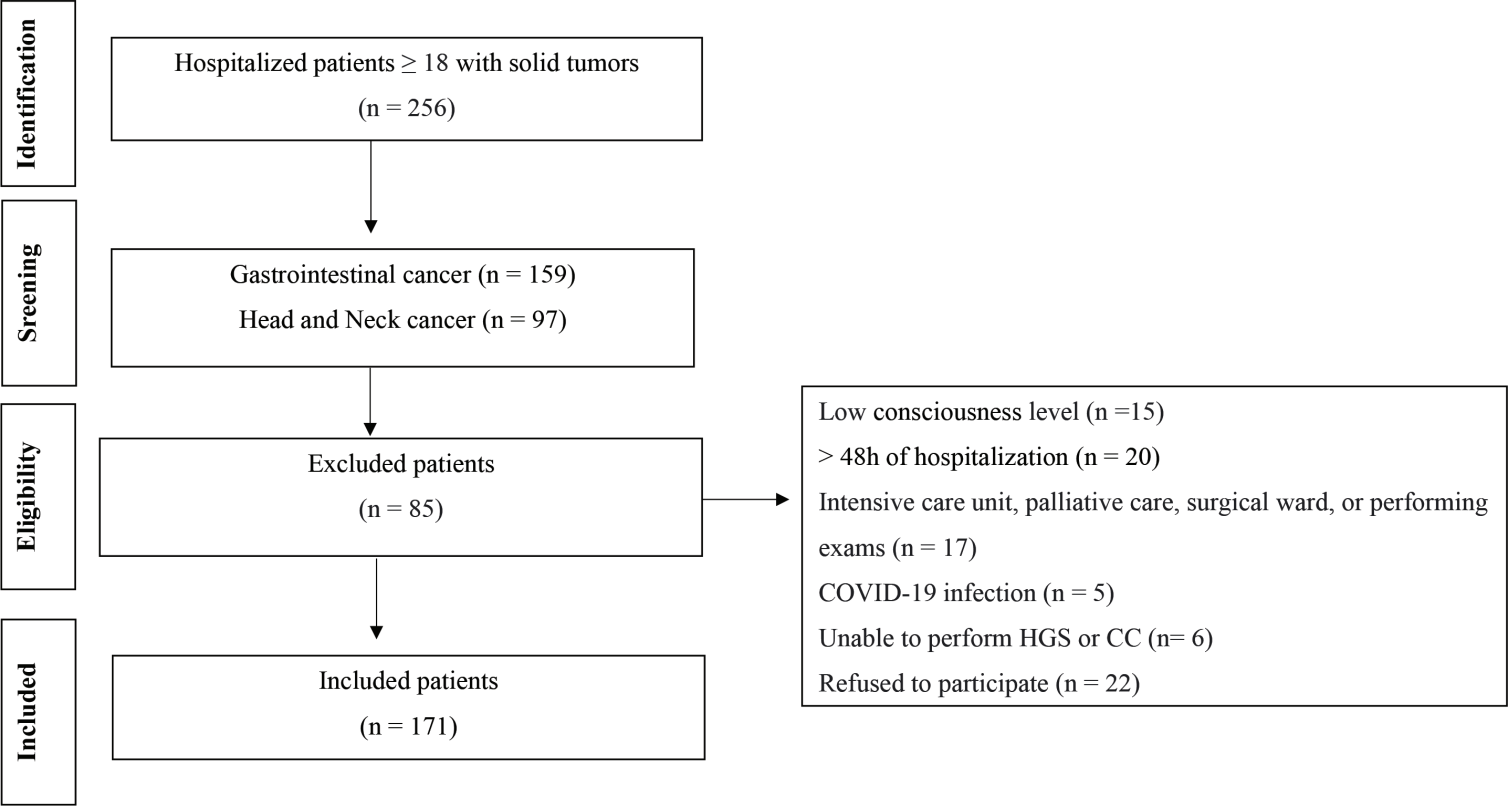
Flowchart of patient selection.

The data analysed in this study are part of a previous cohort study, including patients with different types of cancer admitted to a teaching hospital from May 2021 to March 2022 [36]. The research was prepared in accordance with the Declaration of Helsinki and approved by the Ethics Commission of the Hospital de Clínicas de Porto Alegre (protocol #2019.0708), and each study participant provided written informed consent before data collection. The inclusion criteria were age ≥18 years, a diagnosis of gastrointestinal or head and neck cancer, ability to communicate coherently and intelligibly and ability to undergo an HGS test and CC measurement. Patients in the emergency department, in the intensive care unit, receiving palliative care, or with COVID-19 were excluded. Figure 1 shows a flowchart of the patient selection process.

### Data collection

A trained researcher collected patient data at the bedside within 48 hours of hospital admission. The researcher also reviewed electronic medical records to collect sociodemographic data (e.g., age and sex) and clinical characteristics (e.g., cancer type and stage, metastases, chronic diseases and treatment). Ethnicity was self-reported by the patient or a family member on hospital admission. Self-reported physical activity was obtained by asking patients the following question: ‘Are you engaged in any kind of physical activity?’ (yes/no); if yes, the patients were also asked: ‘What kind of physical activity?’; ‘How many times a week do you usually do this activity?’ and ‘How long have you been doing this activity?’.

All patients were followed until discharge for the assessment of LOS, in-hospital mortality and hospital readmission (within 30 days). Prolonged LOS was defined as LOS ≥6 days (this categorization was based on median values).

### Nutritional assessment and nutritional indicators

Trained researchers performed nutritional assessment and calculated nutritional indicators for each study participant within 48 hours of hospital admission.

### Nutritional assessment

Patients were screened for nutritional risk with the PG-SGA Short Form (PG-SGA SF), consisting of four sections to be completed by the patient: (1) weight history, (2) food intake, (3) nutrition impact symptoms and (4) physical function. The four scores are summed and a total score ≥4 is indicative of nutritional risk and <4 is of no risk [37]. Both SGA and PG-SGA were used to diagnose malnutrition. SGA is considered the reference for nutritional diagnosis and assessment of weight loss, food intake changes, gastrointestinal symptom presence, functional capacity alterations and muscle and subcutaneous fat loss. This tool classifies patients as A, B or C, indicating well nourished, moderately or suspected malnourished and severely malnourished, respectively [19]. The PG-SGA is an oncology-specific tool consisting of two components: (1) patient-generated component, which corresponds to sections 1–4 of the PG-SGA SF, and (2) professional component, to be completed by the researcher, which includes providing information on patients’ history (weight, dietary intake, symptoms of nutrition impact, physical function and metabolic stress) and physical assessment (body fat, muscle mass and fluid retention) [22]. After the assessments were completed, the individuals were categorized as well nourished (category A), moderately or suspected malnourished (category B) or severely malnourished (category C) [22].

### Nutritional indicators

The nutritional indicators evaluated in this study were BMI, HGS and CC. BMI was calculated by dividing the patient’s weight (kg) by height squared (m^2^) and classified according to the World Health Organization (WHO) criteria [30] as low BMI (<18.5 kg/m^2^, underweight), normal BMI (18.5–24.99 kg/m^2^, normal weight) and high BMI (≥25 kg/m^2^, overweight). These classifications were transformed into binary categorical variables (low BMI/high BMI) for analysis.

Patients were tested for dominant hand HGS (kg) with a previously calibrated Jamar^®^ hand dynamometer. In the sitting position and comfortably holding the dynamometer in the dominant hand, with the arm resting at a right angle with the forearm, patients were instructed to squeeze the handle as hard as possible for at least 3 seconds. The best of three attempts, with a 60-second interval between them, was used as maximal muscle strength [38]. Low HGS was defined as low muscle strength interpreted according to the following cutoffs: <27 kg for men and <16 kg for women [25].

With the patient in the sitting position with the legs at a right angle with the thigh, CC was measured at the point of maximum circumference using a Sanny^®^ tape measure. To remove adiposity confounders, CC values were adjusted for BMI by subtracting 3 cm (BMI: 25–29.9 kg/m^2^) or 7 cm (BMI: 30–40 kg/m^2^) from the CC measurement [39]. Low CC was defined as muscle loss interpreted according to the following cutoffs: ≤34 cm for men and ≤33 cm for women [40].

### Statistical analysis

This study is a second part of a cohort study that included patients with solid tumours [36].

The Kolmogorov–Smirnov test assessed the normality of the data. Continuous variables were presented as mean (SD) or median (25th–75th percentiles), whereas categorical variables were presented as counts and percentages.

The accuracy of each nutritional indicator (BMI, HGS and CC) in diagnosing malnutrition was measured by the area under the receiver operating characteristic curve (AUC) compared with the reference standards (SGA and PG-SGA), using sensitivity, specificity, positive predictive value (PPV) and negative predictive value (NPV) with their 95% CIs. Based on the AUC value, the diagnostic accuracy was defined as follows: 0.5–0.6, very poor; 0.6–0.7, poor; 0.7–0.8, moderate; 0.8–0.9, good and >0.9, excellent [41]. Sensitivity and specificity were satisfactory if their values exceeded 80% [42]. The kappa coefficient was calculated to measure the degree of agreement between the nutritional indicators and the reference standards [43]. The results were interpreted as follows: kappa <0, no agreement; 0–0.19, poor agreement; 0.20–0.39, fair agreement; 0.40–0.59, moderate agreement; 0.60–0.79, substantial agreement and 0.80–1.00, almost perfect agreement [43].

Multiple logistic regression analysis was used to determine whether malnutrition was associated with prolonged LOS (≥6 days) as the dependent variable by calculating the odds ratio and 95% CI. Patients categorized in the SGA and PG-SGA as being moderately or severely malnourished were grouped together as having malnutrition. The most important covariates were identified by stepwise regression based on their independent contributions to the models, which were adjusted for adjusted age, sex, ethnicity, type of cancer, presence of metastasis and chronic diseases.

Data were analysed using MedCalc, version 20.116 (MedCalc Software, Mariakerke, Belgium), and SPSS, version 25.0 (IBM, Chicago, IL, USA). The statistical significance was set at *p*-value <0.05.

## Results

### General and clinical results and outcomes

We evaluated 171 patients with a mean age of 61.9 (SD, 12.9) years. Older adults accounted for 64.3% of the sample; 52.0% were men, 87.7% self-identified as White and 83% were physically inactive. Gastrointestinal cancer (gastric and colorectal) accounted for 57.9% of the cases (*n* = 99) and head and neck cancer (mouth, tongue, larynx and pharynx) for 42.1% (*n* = 72). The main reasons for hospitalization are for oncological treatment: 58.5% (*n* = 100) were treated surgically, 8.8% (*n* = 15) received chemotherapy, 1.8% (*n* = 3) were treated with radiotherapy and 23.4% (*n* = 40) received combined treatment. The cancer was diagnosed at an advanced stage (III or IV) in 33.3% of patients (*n* = 57), and 26.9% (*n* = 46) had metastases. Chronic diseases included hypertension (50.3%), diabetes (21.1%) and cardiovascular disease (12.3%). The median LOS was 6 (3–11) days; 56.7% were hospitalized for ≥6 days. Regarding hospital readmission, 20.5% of patients were readmitted within 30 days of discharge. The average in-hospital mortality rate was 7% (*n* = 12). Table 1 shows these results.

### Prevalence of nutritional risk, malnutrition and nutritional indicators

**Figure 2.**
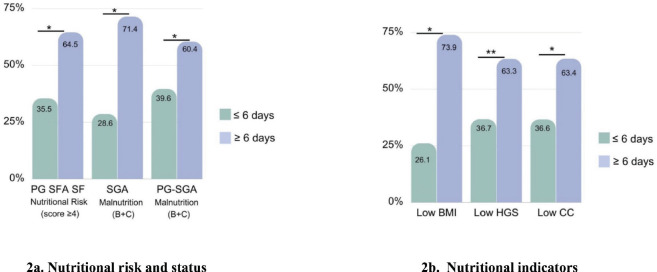
Relation between nutritional characteristics and hospitalization (≥6 days) in patients with gastrointestinal and head and neck cancer (n = 171). (a): Nutritional risk and status. (b): Nutritional indicators. PG-SGA SF, Patient-generated subjective global assessment short form; SGA, subjective global assessment; PG-SGA, patient-generated subjective global assessment; BMI, body mass index; HGS, hand grip strength; CC, calf circumference. ^a^ Chi-square test. ^*^p < 0.001.

Table 2 describes participants’ nutritional characteristics. According to the PG-SGA SF, 72.5% of patients (*n* = 124) were at nutritional risk. Overall, malnutrition (moderately [B] and severely [C] malnourished) was diagnosed in 57.4% of patients with the SGA and 87.2% of patients with the PG-SGA. Regarding nutritional indicators, 59.1% of patients had low CC, 46.2% of patients had low HGS and 13.5% of patients had low BMI. In addition, patients at nutritional risk, with malnutrition, low BMI and low CC, were hospitalized longer than the remaining patients (Figure 2). In this group, the main nutrition impact symptoms reported by the patients were loss of appetite (56.2%), xerostomia (25.1%), nausea (24%) and constipation (19.3%).

### Accuracy of each nutritional indicator in diagnosing malnutrition

**Figure 3.**
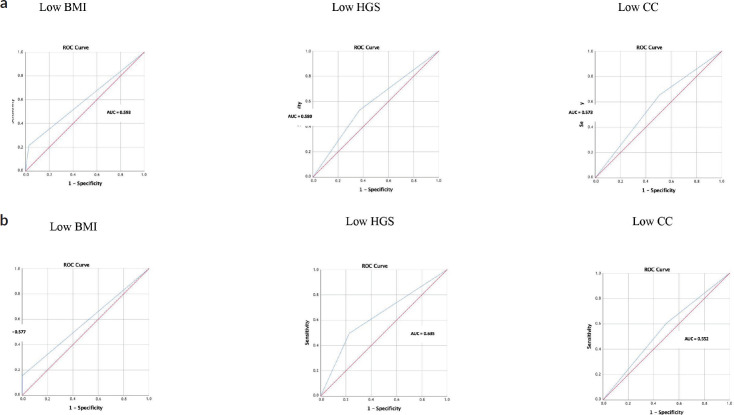
Receiver operating characteristic (ROC) curves using nutritional indicators to diagnose malnutrition in patients with gastrointestinal and head and neck cancer (SGA and PG-SGA as reference methods). (a): SGA as a method reference. (b): PG-SGA as a method reference. AUC, area under curve; BMI, body mass index; HGS, hand grip strength; CC, calf circumference; SGA, subjective global assessment; PG-SGA, patient-generated subjective global assessment.

Table 3 shows how accurately each nutritional indicator (BMI, HGS and CC) can diagnose malnutrition, using SGA and PG-SGA as the reference standard methods (Figure 3). All nutritional indicators performed poorly in diagnosing malnutrition (AUC < 0.70) and were only in fair agreement with SGA and PG-SGA (kappa < 0.40). However, despite a sensitivity of only 21.4%, low BMI had the highest specificity (97.3%) and PPV (91.3%) compared with the other nutritional indicators.

### Association between nutritional indicators and malnutrition as a predictor of prolonged hospitalization

Table 4 describes the association of nutritional indicators (BMI, HGS and CC) and malnutrition (SGA and PG-SGA) with prolonged LOS (≥6 days). The logistic regression model, adjusted age, sex, ethnicity, type of cancer, presence of metastasis and chronic diseases showed that patients with low BMI and low CC were 1.79 (*p * = 0.026) and 1.71 (*p *= 0.111) times more likely to be hospitalized for ≥6 days, respectively. Furthermore, malnourished patients, as diagnosed by SGA and SGA-PG, were 3.60 (*p* < 0.001) and 2.78 (*p *= 0.048) times more likely to have prolonged LOS, respectively, than well-nourished patients.

## Discussion

In this study, we showed that individual nutritional indicators (BMI, HGS and CC) had poor performance (AUC < 0.70) and agreement (kappa < 0.20) in diagnosing malnutrition compared with SGA and PG-SGA. Low BMI (<18.5 kg/m^2^), however, had adequate PPV and specificity (>80%) for the diagnosis of malnutrition. Therefore, the BMI cutoff of 18.5 kg/m^2^ could serve as a complement to nutritional assessment in patients with gastrointestinal or head and neck cancer. Our results also showed a significant association between low BMI and LOS ≥ 6 days (*p* = 0.026).

### Prevalence of nutritional risk, malnutrition and nutritional indicators

Malnutrition is highly prevalent in hospitalized patients with cancer, varying in degree depending on the location and stage of the tumour [44]. In our cohort of patients with gastrointestinal or head and neck cancer, nutritional risk was identified in 72.5% (PG-SGA SF) and malnutrition was identified in 57.4% (SGA) and 87.2% (PG-SGA). Our results are consistent with those of previous studies investigating these two types of cancer, with malnutrition ranging from 43.8% to 86.3% depending on the nutritional assessment tool used [9, 45–47]. In fact, PG-SGA can identify a higher prevalence of malnutrition because it is an oncology-specific tool that assesses symptoms and clinical conditions specific to patients with cancer [22].

Individual nutritional indicators (BMI, HGS and CC) can complement nutritional assessment and have been associated with negative outcomes in patients with cancer [32, 47]. In our study, low BMI according to the WHO criteria [30] was identified in 13.5% of patients, and this group had longer LOS than patients with normal BMI. HGS assesses muscle function and has recently been recommended as a complementary measure in hospitalized patients [17]. Almost half of our patients (46.2%) had low HGS, which agrees with previous studies in which low HGS was identified in 38% of patients with advanced cancer [21] and in 48% of malnourished patients with cancer [48]. Also, having low HGS was found to decrease the odds of hospital discharge by 3-fold compared with having high HGS [23]. CC is an indicator of muscle mass [27], and low CC was identified in 59.1% of our patients after adjusting these values for BMI [39]. Likewise, a prevalence of low CC of 56% was reported in patients with gastric and colorectal cancer [15]. Moreover, in patients with and without cancer, reduced CC values were associated with mortality [28] and hospital readmission [33].

### Accuracy of each nutritional indicator in diagnosing malnutrition

Only low BMI (<18.5 kg/m^2^) had adequate specificity for the diagnosis of malnutrition compared with SGA and PG-SGA. In patients with gastric and colorectal cancer, low BMI (compared with SGA) also demonstrated satisfactory specificity [15].

Low HGS had a high PPV for the diagnosis of malnutrition compared with PG-SGA. Because malnourished patients with cancer are functionally impaired due to loss of muscle function, low HGS may anticipate the detection of nutritional losses, even before the detection of changes in body composition is possible [40]. However, in our study, HGS alone could not diagnose malnutrition. A previous study of patients with cancer showed an association between reduced HGS and malnutrition, which may be explained by the ability of cancer-induced nutritional damage to accelerate the decrease in HGS [48].

CC is closely related to whole-body muscle mass and offers prognostic value for clinical and oncological outcomes [27, 28]. In our study, low CC agreed poorly with SGA and PG-SGA in the diagnosis of malnutrition. Fair agreement has also been found between low CC (<31 cm as the cutoff) and PG-SGA in older patients with cancer [28]. We highlight that, in the present study, we used cutoffs according to sex [40] and adjusted CC for BMI, thus avoiding adiposity confounders [39]. This adjustment is appropriate because it can minimize classification errors, such as classifying patients with obesity as having normal CC values. For example, in the present study, approximately 40% of patients had high BMI.

### Association of nutritional indicators and malnutrition with hospitalization

Patients with low BMI and CC had longer LOS in our study. These data are consistent with the results of a meta-analysis suggesting a relationship between BMI < 18.5 kg/m^2^ and LOS in older patients with cancer [31] and a study reporting an association between low CC and prolonged LOS (≥9 days) in hospitalized adults [49]. In the present study, in addition to a positive association between low BMI and LOS ≥6 days, we found that malnourished patients diagnosed by SGA and PG-SGA were 3.60 and 2.78 times more likely to have prolonged LOS, respectively, than well-nourished patients. In patients with different types of cancer, poorer nutritional status has been associated with prolonged LOS [20, 21, 23, 24].

### Implications for clinical practice

BMI, HGS and CC are widely used in clinical practice, but our findings suggest that these nutritional indicators might inappropriately diagnose malnutrition when used alone in the cancer setting. In patients with gastrointestinal or head and neck cancer, however, BMI < 18.5 kg/m^2^ may be used in combination with SGA or PG-SGA for nutritional assessment. This information has great utility in the clinical setting because BMI values can differ between patient groups [15, 50, 51]. HGS is a measure used to assess muscle function that can complement nutritional assessment and is associated with reduced functional capacity and malnutrition [48], a scenario further complicated in older patients [52]. Approximately 65% of the patients in our sample were older adults. CC is considered a sensitive anthropometric index to assess muscle mass and an important measure to evaluate loss of muscle mass [27], but the use of BMI-adjusted cutoffs has been suggested [39].

This study used PG-SGA, an oncology-specific tool, as a reference to evaluate the diagnostic accuracy of the nutritional indicators. The use of this tool in our population was important because it evaluates signs and symptoms specific to patients with cancer [22]. For instance, 56.2% of our patients reported loss of appetite, which directly affects their nutritional status. Also, LOS may negatively affect patients’ nutritional status; in the present study, malnourished patients had longer LOS than well-nourished patients regardless of the tool used for diagnosis.

Finally, to fully understand the balance between cancer-related metabolic changes and hospital-provided nutrition, more data are required on how different hospitals handle nutritional care and how these interventions influence clinical outcomes. Further studies should focus on the comparative effects of optimized hospital nutrition and the impact of cancer metabolism on malnutrition.

### Limitations

Limitations of this study include heterogeneous ages and cancer stages in our sample, but their effects were minimized by adjusting the logistic regression model for adjusted age, sex, ethnicity, type of cancer, presence of metastasis and chronic diseases. However, our data support that malnutrition is highly prevalent among patients with gastrointestinal or head and neck cancer upon hospital admission, making early nutritional assessment even more important to help mitigate poor clinical outcomes, such as prolonged LOS. Another limitation of our study is that we did not assess the adequacy of hospital food or dietary support provided to patients. Although our primary focus was to assess the performance of isolated nutritional indicators compared with validated tools, we recognize that the quality and adequacy of hospital food can have a significant impact on patient nutritional status. Even though nutritional assessment was performed within the first 48 hours of hospital admission, this limitation should be considered when interpreting the findings, as malnutrition may be influenced by factors beyond those captured by indicators and assessment tools. Future research should further explore the relationship between hospital dietary support and malnutrition to provide a more comprehensive understanding of malnutrition in hospitalized patients with cancer.

## Conclusion

Our findings demonstrated that low BMI (<18.5 kg/m^2^), low HGS (<27 kg for men and <16 kg for women) and low CC (≤34 cm for men and ≤33 cm for women) were nearly accurate in diagnosing malnutrition in patients with gastrointestinal or head and neck cancer compared with SGA (general tool) and PG-SGA (oncology-specific tool) as the reference standards. However, because low BMI was found to be an independent predictor of longer LOS in our sample, it may be used as a complement to the nutritional assessment performed with SGA and PG-SGA in these cancers.

## Conflicts of interest

No conflicts of interest exist.

## Consent to participate

All participants provided their written informed consent.

## Research ethical approval

This research was prepared in accordance with the Declaration of Helsinki and approved by the Ethics Commission of the Hospital de Clínicas de Porto Alegre (protocol #2019.0708).

## Study concept and design

Study concept and design: TS, MSC. Project Management: TS. Data collection: CHS, MSC, GPS and LMS. Data analysis and interpretation: CHS, MSC and GPS. Manuscript writing: CHS and TS. Manuscript review and editing: all authors. Final approval of manuscript: all authors.

## Data availability

The dataset generated in this study is not publicly available.

## Figures and Tables

**Table 1.** Characteristics of patients with gastrointestinal and head and neck cancer (*n* = 171).

Characteristics	Values
General	
Age (years)	63 (54–72)
Older adults (≥60 years)	110 (64.3%)
Sex (male)	89 (52%)
Ethnicity (white)	150 (87.7%)
Physical activity (no)	142 (83%)
Prevalence of type of cancer	
Gastrointestinal	99 (57.9%)
Gastric	43 (25.2%)
Colorectal	56 (32.7%)
Head and neck	72 (42.1%)
Mouth	37 (21.6%)
Tongue	15 (8.8%)
Larynx	11 (6.4%)
Pharynx	9 (5.3%)
Treatment of cancer^*^	
Surgery	100 (58.5%)
Chemotherapy	15 (8.8%)
Radiotherapy	3 (1.8%)
Combined treatment	40 (23.4%)
Tumor stage III/IV	57 (33.3%)
Presence of metastasis (yes)	46 (26.9%)
Chronic diseases	
Hypertension	86 (50.3%)
Type 2 diabetes	36 (21.1%)
Cardiovascular disease	21 (12.3%)
Outcomes	
LOS (days)	6 (3–11)
≥6 days	97 (56.7%)
Readmission in 30 days (yes)	35 (20.5%)
Death (yes)	12 (7%)

Data expressed as *n* (%) or median (IQR)

* Note: *n* = 13 patients awaiting cancer treatment at the time of data collection

**Table 2.** Nutritional characteristics of patients with gastrointestinal and head and neck cancer (*n* = 171).

Risk and nutritional status	Values
Nutritional risk by PG-SGA SF (score ≥ 4)	124 (72.5%)
Malnutrition by SGA	
Well nourished (A)	73 (42.7%)
Moderately nourished (B)	69 (40.4%)
Severely malnourished (C)	29 (17%)
Malnutrition by PG-SGA	
Well nourished (A)	22 (12.9%)
Moderately nourished (B)	55 (32.2%)
Severely malnourished (C)	94 (55%)
Nutritional indicators	
BMI (kg/m^2^)	26.2 (5–41)
Low BMI^a^	23 (13.5%)
Normal BMI	74 (43.3%)
High BMI	74 (43.3%)
Low HGS (kg)^b^	79 (46.2%)
Low CC (cm)^c^	101 (59.1%)
Treatment symptoms and nutritional effects	
Appetite loss	96 (56.2%)
Xerostomia	43 (25.1%)
Nausea	41 (24%)
Constipation	33 (19.3%)

PG-SGA SF, Patient-Generated Subjective Global Assessment Short Form; SGA, Subjective Global Assessment; PG-SGA, Patient-Generated Subjective Global Assessment; BMI, Body Mass Index; HGS, hand grip strength; CC, calf circumference

Data expressed as *n* (%) and median (p25-p75)

a Low BMI: according to WHO (<18.5 kg/m^2^) [23]

b Low HGS: Male (<27 kg); Female (<16 kg) [18]

c Low CC: Male (≤34 cm); Female (≤33 cm) [33] CC values were adjusted by patients’ BMI to remove the confounding effects of adiposity [32]

**Table 3.** Accuracy of isolated nutritional indicators (BMI, HGS and CC) in diagnosing malnutrition in patients with gastrointestinal and head and neck cancer (using SGA and PG-SGA as reference methods) (n = 171).

Nutritional indicators			
	Low BMI^a^	Low HGS^b^	Low CC^c^
**SGA as reference**			
Kappa (*p*-value)	0.165 (*p* < 0.001)^*^	0.156 (*p* = 0.037)^**^	0.147 (*p* = 0.054)
Accuracy (%)	53.8	57.3	58.5
AUC ROC (CI 95%)	0.593 (0.509–0.678)	0.580 (0.494–0.667)	0.573 (0.486–0.660)
Sensitivity (%)	21.4	53.1	65.3
Specificity (%)	97.3	63.0	49.3
PPV (%)	91.3	65.8	63.4
NPV (%)	47.9	50.0	51.4
PG-SGA as reference			
Kappa (*p*-value)	0.045 (*p* = 0.048)^**^	0.114 (*p* = 0.018)^**^	0.054 (*p* = 0.354)
Accuracy (%)	26.3	53.2	59.1
AUC ROC (CI 95%)	0.577 (0.462–0.692)	0.635 (0.517–0.752)	0.552 (0.422–0.682)
Sensitivity (%)	15.4	49.7	60.4
Specificity (%)	100.0	77.3	50.0
PPV (%)	100.0	93.6	89.1
NPV (%)	14.9	18.58	15.8

Abbreviations: BMI, Body Mass Index; HGS, hand grip strength; CC, calf circumference; SGA, Subjective Global Assessment; PG-SGA, Patient-Generated Subjective Global Assessment

SGA: 57.3% malnutrition prevalence.

PG-SGA: 87.1% malnutrition prevalence.

a Low BMI: according to WHO (<18.5 kg/m^2^) [23]

b Low HGS: Male (<27 kg); Female (<16 kg) [18]

c Low CC: Male (≤34 cm); Female (≤33 cm) [33]CC values were adjusted by the patient’s BMI, to help to remove the confounding effects of adiposity [32]

* *p* < 0.001;

** *p* < 0.05.

**Table 4.** Nutritional indicators and malnutrition associated with hospitalization (≥6 days) in patients with gastrointestinal and head and neck cancer: logistic regression model (*n* = 171).

	OR^a^	95%CI	*p*-value
Nutritional indicators			
Low BMI^b^	1.79	1.64–5.00	0.026
Low HGS^c^	1.53	0.77–3.02	0.218
Low CC^d^	1.71	0.88–3.31	0.111
Malnutrition			
SGA (B and C)^e^	3.60	1.83–7.09	<0.001^*^
PG-SGA (B and C)^e^	2.78	1.01–7.67	0.048^**^

OR, Odds Ratio; CI, Confidence Interval; BMI, Body Mass Index; HGS, Hand Grip Strength; CC, Calf Circumference; SGA, Subjective Global Assessment; PG-SGA, Patient-Generated Subjective Global Assessment

a Models were adjusted by age, sex, presence of metastasis, and chronic diseases

b Low BMI: according to WHO (<18.5 kg/m^2^) [23]

c Low HGS: Male (<27 kg); Female (<16 kg) [18]

d Low CC: Male (≤34 cm); Female (≤33 cm) [33] CC values were adjusted by patients’ BMI to remove the confounding effects of adiposity [32]

e Malnutrition by SGA and PG-SGA: patients classified as moderately (B) and severely malnourished (C) were grouped

* *p* < 0.001;

** *p* < 0.05
